# Risk Factors of Metabolic Syndrome Among Patients Receiving Antipsychotics: A Retrospective Study

**DOI:** 10.1007/s10597-019-00537-y

**Published:** 2019-12-28

**Authors:** Samer Hammoudeh, Hawra Al Lawati, Suhaila Ghuloum, Huma Iram, Arij Yehya, Imen Becetti, Nora Al-fakhri, Hany Ghabrash, Mena Shehata, Nighat Ajmal, Iman Amro, Hira Safdar, Yassin Eltorki, Hassen Al-Amin

**Affiliations:** 1 Research Department, Weill Cornell Medicine - Qatar, Doha, Qatar; 2 Medical Education Department, Weill Cornell Medicine - Qatar, Doha, Qatar; 3 Psychiatry Department, Mental Health Hospital, Hamad Medical Corporation, Doha, Qatar; 4 Pharmacy Department, Rumailah Hospital, Hamad Medical Corporation, Doha, Qatar; 5 Psychiatry Department, Weill Cornell Medicine - Qatar, Education city, P.O. Box: 24144, Doha, Qatar

**Keywords:** Metabolic syndrome, Antipsychotics, Arabs, Mental health, Retrospective, Qatar

## Abstract

This study aimed to assess the differential effects of first-generation (FGA) and second-generation antipsychotics (SGA) on the prevalence of risk factors for metabolic syndrome among mentally ill patients in Qatar. We also wanted to check if there is proper adherence with the guidelines for prescribing antipsychotics and the monitoring of metabolic effects in this population. We collected the available retrospective data (socio-demographic, psychiatric, anthropometric, and metabolic measures) from the records of 439 patients maintained on antipsychotics. The majority were males, married, employed, having a psychotic disorder, and receiving SGA. Patients on SGA showed more obesity, higher BP, and more elevated triglycerides compared to those on FGA. The prevalence of the abnormal metabolic measures was high in this sample, but those on SGA showed a significantly higher prevalence of abnormal body mass index and BP. Obesity and hypertension were common in patients maintained on antipsychotics, especially those on SGA. Polypharmacy was common, and many metabolic measures were not monitored properly in those maintained on antipsychotics. More prospective studies with guided monitoring of the patients' clinical status and metabolic changes are needed to serve better this population of patients.

## Introduction

Psychosis is a common mental condition that might alter the reality testing of the affected individuals (Kay and Tasman 2006). Psychotic disorders include schizophrenia, schizophreniform, schizoaffective disorder, brief psychotic disorder, substance/medication related psychotic disorders, a catatonic disorder due to other medical conditions, and delusional disorder (Black and Andreasen 2014). These disorders come with a high burden for both the patient and society (Radua et al. 2018; Rössler et al. 2005; van Os and Kapur 2009). Antipsychotics (AP) have been indicated for the treatment of schizophrenia spectrum disorders (Citrome 2013; Sagud et al. 2013), mood disorders with psychotic features (Singh et al. 2012), bipolar disorder (Hirschfeld et al. 2010), and psychosis in general (Jethwa and Onalaja 2015). Metabolic syndrome (MetS) is a group of abnormalities that have been shown to increase the risk of cardiovascular disease (Mottillo et al. 2010) and thus increase morbidity and mortality (Vancampfort et al. 2015). These abnormalities include obesity, increased triglycerides (TG), low high-density lipoprotein (HDL), increased blood pressure (BP), and increased fasting glucose (Mottillo et al. 2010). Numerous studies reported higher rates of morbidity and risk of mortality among patients with psychosis (Bobes et al. 2012; Feng and Melkersson 2012; Nuevo et al. 2011; Sugawara et al. 2010). These high rates were attributed to the higher prevalence of metabolic abnormalities in this population. However, it is still not clear how much the psychosis itself or the use of AP contributes to these metabolic effects.

An international health survey conducted by World Health Organization showed that the risk of having diabetes mellitus increases with the presence of psychotic symptoms, even after controlling for other confounders like diagnosis and previous intake of AP (Nuevo et al. 2011). Other population studies reported that there were no differences in the prevalence of MetS between patients with chronic mental illness and controls (Baptista et al. 2011; Suzuki et al. 2013; Saloojee et al. 2016). On the other hand, studies have linked the intake of AP with a higher risk of developing or worsening of MetS risk factors (Centorrino et al. 2012; De Hert et al. 2011; Kagal et al. 2012; Misiak et al. 2014; Rojo et al. 2015; Vancampfort et al. 2015). Different AP can have variable effects on metabolic abnormalities. For example, clozapine and olanzapine were associated with a substantial increase in weight when compared to other second-generation antipsychotics (SGA), while chlorpromazine was the worst culprit amongst the first-generation antipsychotics (FGA) (De Hert et al. 2011). However, few studies from other countries like the one from Venezuela (Baptista et al. 2011) reported that there were no differences in MetS when comparing patients on AP and the general population.

Thus, it seems that ethnicity and culture might variably affect the metabolic changes associated with the intake of AP for various psychiatric disorders. A recent study in Qatar reported a MetS prevalence rate of 31.9% and 22.8% among patients maintained on AP and the general population, respectively (Hammoudeh et al. 2018). An earlier study in Qatar reported a MetS prevalence rate of 36.5% and 18.7% among schizophrenia patients and healthy controls, respectively (Bener et al. 2014). In the Arabic Peninsula, few studies have addressed the relationship between mental disorders, type of AP, and metabolic changes. In the United Arab Emirates (UAE), it was reported that the prevalence of MetS is 58.9% and 44.2% for schizophrenia and bipolar patients, respectively (Shahda et al. 2010). A Kuwaiti study reported a MetS prevalence rate of 27.1% among schizophrenia patients (Roshdy 2011). In Palestine, a prevalence rate of 43.6% was reported among 250 schizophrenia patients (Sweileh et al. 2012). Most of these studies addressed the associations of MetS with mental illness but not the impact of various AP on the different metabolic parameters. Thus, our main objective was to retrospectively assess the differences in the metabolic profile in patients receiving only FGA, only SGA, or a combination of both in the population of Qatar. We hypothesized that there are significant differences in MetS risk factors among the different types of AP. We also wanted to check the frequency of polypharmacy and whether the monitoring of the metabolic effects in these patients taking AP was adequate.

## Methods

This retrospective chart review was part of a larger project investigating the biopsychosocial aspects related to psychotic disorders in Qatar. This paper focuses on the section assessing the metabolic abnormalities in patients maintained on AP. The study was conducted between June 2014 and June 2016. The ethics committees at the Medical Research Center of Hamad Medical Corporation (HMC), Doha, Qatar, and Weill Cornell Medicine in Qatar (WCM-Q) granted ethical approvals. Using convenience sampling, we retrieved the records of patients (age above 18 years) who were receiving AP at the Psychiatry Hospital in Doha, during the period between October 2012 and April 2014. We found a total of 645 cases. However, we reviewed only 439 files because we excluded the cases that received AP for periods less than three months or in small doses (< 50 mg equivalent of chlorpromazine, mainly to target agitation, anxiety, or insomnia). The chlorpromazine equivalent AP dose was calculated using the method by Andreasen et al. (2010). There were no other exclusion criteria. The Psychiatry Hospital that is run by the Department of Psychiatry, HMC is the only psychiatric facility in Qatar. It has 70 inpatient beds (average occupancy 95%; the majority is admitted with acute psychosis or mania) and ten outpatient clinics with about 120 visits per weekday (about 30% have psychotic disorders). It is worth adding that Qatar is a developing country with rapid economic growth where the majority of the population are expatriates from the South and East Asia. The Qataris and Arabs constitute only about 28% of the residents in Qatar (total population about 2.5 million in 2015 with male to female ratio of about 3) (Qatar Statistics Authority 2015).

A set of questionnaires were designed to collect the following information: socio-demographics, clinical features related to medical and psychiatric history, medication intake, available anthropometric measures, and laboratory data related to diabetes and lipid profiles. These questionnaires were piloted and validated in a sample of 20 records before collecting the research data. We developed a manual to be used by all raters to code the data retrieved from the records and to be used by all raters. Ten raters (one postdoc in public health, one MD, one nurse, one clinical psychologist, three psychiatry residents, and three medical students) were involved in collecting different sections of the questionnaires for this project. All raters received proper training and supervision before collecting the real data. To ensure excellent inter-rater reliability, 100 records were assessed independently by two raters who were blind to the results of each entry. We collected the psychiatric diagnosis from the records according to the treating psychiatrists who mostly use the Diagnostic and Statistical Manual of Mental Disorders (DSM) criteria for diagnosis. All the laboratory results were done by the same laboratory located at General Medical Hospital, HMC, Doha, Qatar. The last available values in the records were included in this study. We determined the cutoffs for the abnormal values on the anthropometric measures and laboratory results according to the National Cholesterol Education Program, Adult Treatment Panel (ATP III) criteria for MetS: Waist circumference above 102 cm and 88 cm, for men and women, respectively; TG ≥ 1.7 mmol/L; HDL cholesterol levels below < 1.04 mmol/L and < 1.30 mmol/L, for men and women, respectively; BP reading of ≥ 130/ ≥ 85 mm Hg; Fasting glucose ≥ 6.1 mmol/L (Grundy et al. 2004). However, the waist circumference was not routinely collected by the staff at that time, and thus we could not determine the rate of MetS in this cohort.

### Statistical Analysis

The Statistical Package for the Social Sciences (IBM-SPSS, version 23.0, IBM Corp, Armonk, NY, USA) was used to run the various analyses. A p-value of less than 0.05 was considered statistically significant. Descriptives are reported as a frequency for categorical variables or a mean and standard deviation (SD) for the continuous ones. The patients were divided according to their use of FGA only (n = 83), SGA only (n = 302), or a combination of both (n = 152). The differences between these groups were analyzed using a chi-square test for categorical variables and one-way analysis of variance (ANOVA) for the continuous ones. We used Fisher's exact and Mann–Whitney tests when parametric assumptions were not fulfilled. Bonferroni correction was used to control for multiple comparisons.

Concerning missing data, we calculated the percentage for each variable and category in the study. The continuous variables with 25% or more of missing data were dropped from the analysis. For the nominal variables, the missing data was added and analyzed as a separate category. When the latter was more than 25%, we did not proceed with the interpretation of the results. Furthermore, if the missing data category was showing significance, then the results were considered invalid. We did a thorough and careful confirmation of the collected data before any of the above analyses.

## Results

### Sociodemographic Characteristics by Type of Antipsychotics

The majority of the participants were males, married, and employed. The mean age of the sample was 33.68 ± 10.17 years. The majority of the sample was on SGA (57.18%), while 12.30% were on FGA, and 30.52% were on a combination of both. The male to female ratio was similar in the three groups. The majority of the SGA group was married, while those in the combination group were single. Non-Arabs had a higher representation in our sample compared to Qataris/Arabs. The percentages of Arabs and Qataris who completed college and were living with their families in Qatar were significantly higher in the groups receiving SGA or a combination of both when compared to those from the FGA group (p < 0.05). Subjects labeled as “others” (see Table 1) showed significantly higher percentage of patients receiving a combination of FGA and SGA or FGA alone than those on SGA alone (p < 0.05).

**Table 1:** Socio-demographic data of the study sample by Antipsychotics group

Variable	A: FGA (N = 54)	B: SGA (N = 251)	C: Combination (N = 134)
Age	32.40 ± 6.95	35.10 ± 11.16	31.56 ± 8.84

n (%)			
Gender			
Male	39 (72.2%)	159 (63.3%)	90 (67.2%)
Female	15 (27.8%)	92 (36.7%)	44 (32.8%)
Nationality^a^			
Qataris/Arabs	10 (18.5%)	120 (47.8%)A	53 (39.6%)A
South Asia	25 (46.3%)	84 (33.5%)	44 (32.8%)
Filipino	5 (9.3%)	21 (8.4%)	11 (8.2%)
Others	14 (25.9%)B	26 (10.4%)	26 (19.4%)B
Marital status			
Married	29 (53.7%)	136 (54.6%)C	47 (35.9%)
Single	21 (38.9%)	98 (39.4%)	75 (57.3%)B
Divorced/widowed	4 (7.4%)	15 (6.0%)	9 (6.9%)
Education			
None	3 (6.1%)	11 (4.6%)	4 (3.2%)
School	18 (36.7%)	86 (35.7%)	55 (44.0%)
Vocational	02 (.0%)	4 (1.7%)	1 (.8%)
College	1 (2.0%)	47 (19.5%)A	20 (16.0%)A
Missing	27 (55.1%)	93 (38.6%)	45 (36.0%)
Work status			
Employed	34 (63.0%)	123 (49.0%)	65 (48.5%)
Not employed	14 (25.9%)	74 (29.5%)	37 (27.6%)
Other	6 (11.1%)	54 (21.5%)	32 (23.9%)
Living condition			
Co-workers	14 (25.9%)B	26 (10.4%)	19 (14.2%)
Alone	0 (.0%)	9 (3.6%)	6 (4.5%)
Sponsor	6 (11.1%)	12 (4.8%)	16 (11.9%)B
Family	15 (27.8%)	149 (59.4%)A	71 (53.0%)A
Others	9 (16.7%)C	23 (9.2%)	7 (5.2%)
Missing	10 (18.5%)	32 (12.7%)	15 (11.2%)
Having family in Qatar			
Yes	17 (31.5%)	160 (64.0%)A	76 (57.1%)A
No	24 (44.4%)B,C	42 (16.8%)	32 (24.1%)
Missing	13 (24.1%)	48 (19.2%)	25 (18.8%)

For each significant pair, the key of the smaller category appears in the category with the larger proportion. Significance level for upper case letters (A, B, C): 0.05

^a^Arab: Algeria, Bahrain, Egypt, Iraq, Jordan, Lebanon, Mauritania, Morocco, Oman, Palestine, Qatar, Saudi Arabia, Somalia, Sudan, Syria, Tunisia, United Arab Emirates, and Yemen. South Asia: Afghanistan, Bangladesh, India, Indonesia, Iran, Nepal, Pakistan, Sri Lanka, and Thailand. Other: Armenia, Burkina Faso, Canada, China, Czech Republic, Eritrea, Ethiopia, France, Ghana, Ireland, Kenya, Mali, Nigeria, North Korea, South Africa, South Korea, Tanzania, Uganda, United Kingdom, and United States of America

### Diagnosis and Medication Profiles of the Sample by Group of Antipsychotics

The majority of the subjects in each group had a psychotic disorder followed by mood disorder, mainly bipolar disorder (Table 2). There were no significant differences between the groups when comparing the duration of illness and that of the last treatment. However, the 100 mg chlorpromazine equivalent dose was significantly higher in the combination group when compared to the other two groups. In the FGA group, the majority was on haloperidol (n = 42) followed by trifluoperazine (n = 11), chlorpromazine (n = 9), fluphenazine (n = 2), and zuclopenthixol (n = 2). In this group, 15 patients were receiving two FGA. In the SGA group, the majority was on olanzapine (n = 130) followed by Risperidone (n = 83), quetiapine (n = 60), Aripiprazole (n = 20), paliperidone (n = 12), clozapine (n = 7), and amisulpride (n = 2). Thus, 63 patients were using two SGA. In the combination group, the FGA were: haloperidol (n = 52), chlorpromazine (n = 47), trifluoperazine (n = 45), flupenthixol (n = 16), and zuclopenthixol (n = 6); and the SGA were: olanzapine (n = 88), Risperidone (n = 62), quetiapine (n = 20), Aripiprazole (n = 9), paliperidone (n = 7), and clozapine (n = 5). A total of 32 patients were receiving 3 or 4 AP in the combination group.

**Table 2:** Diagnosis and medication profiles by Antipsychotics group

Variable	A: FGA N = 54	B: SGA N = 251	C: Combination N = 134
Mean ± SD			
Duration of illness (yrs.)	2.79 ± 4.49	5.06 ± 7.16	4.50 ± 5.71
Duration of last AP (months)	15.80 ± 6.31	15.69 ± 6.23	15.59 ± 6.03
AP equivalent dose	456.59 ± 441.79	460.60 ± 649.99	839.92 ± 814.79 A,B
n (%)			
Psychotic disorders (total)	*38 (70.37%)*	*174 (69.32%)*	*121 (90.30%)*A,B
Schizophrenia spectrum	32 (84.21%)	157 (90.22%)	103 (85.12%)
NOS	6 (15.79%)	17 (9.78%)	8 (14. 88%)
Mood disorders (total)	*10 (18.52%)*	*58 (23.11%)*	*17 (12.69%)*
Depression	0 (0.0%)	10 (17.24%)	1 (5.88%)
Bipolar	3 (30%)	32 (55.17%)	11 (64.71%)
NOS	7 (70%)	16 (27.59%)	5 (29.41%)
Others (total)	*6 (11.11%)*	*16 (6.37%)*	*3 (2.24%)*
OCD	0 (0.0%)	4 (25.0%)	2 (66.67%)
Substance related	5 (83.33%)	9 (65.25%)	1 (33.33%)
Anxiety disorders	1 (16.67%)	3 (18.75%)	0 (0.0%)
Missing Diagnosis (total)	*0 (0.0%)*	*3 (1.20%)*	*3 (2.24%)*
Other medications (total)	*39 (100%)*	*141 (100%)*	*139 (100%)*
Antidepressants	13 (33.33%)	84 (59.58%)A,C	35 (25.18%)
Benzodiazepines	7 (17.95%)	21 (14.89%)	30 (21.58%)
Anticholinergics	12 (30.77%)B	11 (7.80%)	47 (33.82%)B
Mood Stabilizers	7 (17.95%)	25 (17.73%)	27 (19.42%)

For each significant pair, the key of the smaller category appears in the category with the larger proportion. Significance level for upper case letters (A, B, C): 0.05

*NOS* not otherwise specified, *OCD* obsessive compulsive disorder, *FGA* first generation antipsychotics, *SGA* second generation antipsychotics, *AP* antipsychotic

The numbers in italics show the total number of subjects in each group for the corresponding variable

The proportions of the mood stabilizers and benzodiazepines taken by the patients were not different in the three groups. The mood stabilizers were sodium valproate n = 34, carbamazepine n = 23, lithium n = 1 and lamotrigine n = 1). The percentage of patients receiving anticholinergics was higher in the FGA and combination groups when compared to the SGA group. A higher proportion of patients receiving antidepressants were seen in the SGA group when compared to FGA alone or the combination group. The antidepressants prescribed were mostly selective serotonin re-uptake inhibitors followed by mirtazapine, and about 15 patients were receiving more than one antidepressant.

### Psychiatric Features of the Sample by Group of Antipsychotics

Table 3 compares the symptoms and other psychiatric features of the study sample. The proportion of patients with hallucinations was higher in the combination group compared to the SGA one. Substance use involved mainly alcohol and marijuana, with few cases abusing prescribed benzodiazepines or opiates. However, there was significant missing data in the other categories related to the type of psychotic symptoms and history of suicidal ideation, and thus results could not be appropriately interpreted.

**Table 3:** Psychiatric characteristics of the study sample by Antipsychotics group

Variable N = 439	A: FGA (N = 54) (n%)	B: SGA (N = 251) (n%)	C: Combination (N = 134) (n%)
Delusions			
Yes	17 (31.5)	83 (33.1)	58 (43.3)
No	22 (40.7)	107 (42.6)	46 (34.3)
Missing	15 (27.8)	61 (24.3)	30 (22.4)
Hallucinations			
Yes	27 (50.0)	97 (38.6)	70 (52.2)B
No	19 (35.2)	114 (45.4)C	42 (31.3)
Missing	8 (14.8)	40 (15.9)	22 (16.4)
Disorganized thoughts			
Yes	12 (22.2)	51 (20.3)	38 (28.4)
No	22 (40.7)	134 (53.4)C	53 (39.6)
Missing	20 (37.0)	66 (26.3)	43 (32.1)
Catatonia			
Yes	0 (.0)	5 (2.0)	7 (5.2)
No	16 (29.6)	68 (27.1)	30 (22.4)
Missing	38 (70.4)	178 (70.9)	97 (72.4)
Negative symptoms			
Yes	4 (7.4)	32 (12.7)	17 (12.7)
No	20 (37.0)	55 (21.9)	29 (21.6)
Missing	30 (55.6)	164 (65.3)	88 (65.7)
Suicidal ideation			
Yes	9 (16.7)	34 (13.5)	21 (15.7)
No	33 (61.1)	158 (62.9)	80 (59.7)
Missing	12 (22.2)	59 (23.5)	33 (24.6)
Suicidal attempts			
Yes	6 (11.1)	21 (8.4)	13 (9.7)
No	29 (53.7)	110 (43.8)	58 (43.3)
Missing	19 (35.2)	120 (47.8)	63 (47.0)
Substance use			
Yes	15 (27.8)	50 (19.9)	23 (17.2)
No	30 (55.6)	150 (59.8)	77 (57.5)
Missing	9 (16.7)	51 (20.3)	34 (25.4)

For each significant pair, the key of the smaller category appears in the category with the larger proportion. Significance level for upper case letters (A, B, C): 0.05

*FGA *first generation antipsychotics, *SGA* second generation antipsychotic

### Anthropometric and Metabolic Measures by Antipsychotics Group

ANOVA showed that weight, BMI, systolic BP, and TG measures were significantly different in the three groups. Post hoc comparisons showed that the mean weight and BMI were significantly higher in the SGA group when compared to the FGA one; systolic BP and TG measures were significantly higher in the SGA and the combination groups than the FGA one. Furthermore, the TG was even higher in the combination group when compared to the ones in the SGA group (Table 4). When analyzing the number of subjects with abnormal metabolic measures (Table 5), the number of participants with elevated BP was higher in the SGA and combination groups, and the number of subjects with obesity, as measured by BMI, was the highest in the group taking SGA (Table 5).

**Table 4:** Anthropometric and metabolic measures by Antipsychotics group

Variable (n)	A: FGA (mean ± SD)	B: SGA (mean ± SD)	C: Combination (mean ± SD)	Total (mean ± SD)
Weight (kg)	64.00 ± 18.46 (n = 53)	72.64 ± 19.68 A (n = 230)	68.88 ± 20.45 (n = 123)	70.37 ± 19.94 (n = 406)
BMI (kg/m^2^)	23.30 ± 4.82 (n = 39)	26.88 ± 6.40 A (n = 202)	25.96 ± 12.29 (n = 108)	26.20 ± 8.60 (n = 349)
Systolic BP (mmHg)	123.48 ± 12.27 (n = 54)	128.60 ± 14.35 A (n = 247)	128.92 ± 12.77 A (n = 132)	128.06 ± 13.72 (n = 433)
Diastolic BP (mmHg)	78.17 ± 8.20 (n = 54)	78.65 ± 10.36 (n = 245)	78.74 ± 9.03 (n = 133)	78.62 ± 9.70 (n = 432)
HbA1C	5.47 ± .72 (n = 6)	6.18 ± 2.09 (n = 54)	6.40 ± 2.13 (n = 29)	6.20 ± 2.04 (n = 89)
Fasting glucose (mmol/L)	5.59 ± 1.45 (n = 49)	5.84 ± 2.46 (n = 228)	5.76 ± 3.18 (n = 125)	5.78 ± 2.61 (n = 402)
Cholesterol (mmol/L)	4.48 ± .90 (n = 24)	4.72 ± 1.06 (n = 128)	4.68 ± 1.38 (n = 59)	4.68 ± 1.14 (n = 211)
Triglycerides (mmol/L)	1.06 ± 0.67 (n = 24)	1.35 ± 0.85 A (n = 127)	1.67 ± 1.79 A,B (n = 59)	1.41 ± 1.19 (n = 210)
HDL (mmol/L)	1.14 ± 0.39 (n = 24)	1.27 ± 0.38 (n = 122)	1.18 ± 0.40 (n = 58)	1.23 ± 0.39 (n = 204)
LDL (mmol/L)	2.86 ± 0.70 (n = 24)	2.83 ± 0.87 (n = 121)	2.80 ± 1.18 (n = 58)	2.82 ± 0.95 (n = 203)

For each significant pair, the key of the smaller category appears in the category with the larger mean. Significance level for upper case letters (A, B, C): 0.05

*FGA* first generation antipsychotics, *SGA* second generation antipsychotics, *BMI* body mass index, *BP* blood pressure, *HbA1C* glycosylated hemoglobin, *HDL* high density lipoproteins, *LDL* low density lipoproteins

**Table 5:** Metabolic abnormalities based on ATP III criteria by Antipsychotics group

Variable	A: FGA n (%)	B: SGA n (%)	C: Combination n (%)	Total n (%)
TG				
Not met	20 (83.3)	101 (79.5)	43 (74.1)	164 (78.5)
Met	4 (16.7)	26 (20.5)	15 (25.9)	45 (21.5)
Total	24 (100.0)	127 (100.0)	58 (100.0)	209 (100.0)
HDL (males)				
Not met	9 (47.4)	47 (61.8)	22 (52.4)	78 (56.9)
Met	10 (52.6)	29 (38.2)	20 (47.6)	59 (43.1)
Total	19 (100.0)	76 (100.0)	42 (100.0)	137 (100.0)
HDL (females)				
Not met	3 (60.0)	32 (69.6)	10 (62.5)	45 (67.2)
Met	2 (40.0)	14 (30.4)	6 (37.5)	22 (32.8)
Total	5 (100.0)	46 (100.0)	16 (100.0)	67 (100.0)
BP				
Not met	36 (66.7)B,C	115 (46.6)	55 (41.4)	206 (47.5)
Met	18 (33.3)	132 (53.4)A	78 (58.6)A	228(52.5)
Total	54 (100.0)	247 (100.0)	133 (100.0)	434 (100.0)
Glucose				
Not met	34 (69.4)	169 (74.1)	99 (79.8)	302 (75.3)
Met	15 (30.6)	59 (25.9)	25 (20.2)	99 (24.7)
Total	49 (100.0)	228 (100.0)	124 (100.0)	401 (100.0)
BMI				
Underweight	3 (7.7)	7 (3.5)	6 (5.6)	16 (4.6)
Normal	26 (66.7)B	88 (43.6)	63 (58.3)B	177 (50.7)
Obese	10 (25.6)	96 (47.5)A, C	33 (30.6)	139 (39.8)
Very obese	0 (.0)	11 (5.4)	6 (5.6)	17 (4.9)
Total	39 (100.0)	202 (100.0)	108 (100.0)	349 (100.0)

For each significant pair, the key of the smaller category appears in the category with the larger mean. Significance level for upper case letters (A, B, C): 0.05

*FGA* first generation antipsychotics, *SGA* second generation antipsychotics, *TG* triglycerides, *HDL* high density lipoproteins, LDL low density lipoproteins, *BP* blood pressure, *BMI* body mass index

### Monitoring of Patients on Antipsychotics and Other Psychotropics

Most of the participants were on SGA, but the polypharmacy is evident as more than one-third of the participants were on two AP, and several patients were even on three. There was significant missing clinical information on the psychotic features (Table 3) and no documented justification for this polypharmacy. Again, many subjects in this sample did not have measures of the lipid profile or even BMI, and none had the measure of waist circumference, which are essential measures to monitor in patients maintained on AP (Table 4). Polypharmacy was also evident by the high number of patients on additional benzodiazepines, antidepressants, or mood stabilizers. Patients on SGA only were also receiving anticholinergics (Table 2).

## Discussion

The focus of this study was on investigating the MetS risk factors among patients maintained on SGA, FGA, or combination of both, and whether these patients were monitored properly. The following is a discussion of the sociodemographic, clinical, and metabolic profiles of our sample, and the deficits in the proper documentation and monitoring of patients on AP. Our results will be summarized below and then compared to those in the regional and international studies related to the same objectives.

The mean age of our sample was in the early 30 s, with no differences between the three groups. The majority of participants in each group were males, reflecting the population in Qatar, where the male to female ratio is about 3 (Qatar Statistics Authority 2015). The majority in the SGA and the combination groups were mostly Qataris and Arabs. The latter constitute only about 28% of the residents of Qatar (Qatar Statistics Authority 2015), but they are the most stable population, while single expatriates who need maintenance on AP might leave the country to be with their original support families. This observation would also explain why the majority in this sample was employed and living with their families in Qatar (Table 1). The preponderance of our sample had psychotic disorders followed by bipolar and other mood disorders. The duration of illness and that of the last treatment regimen were not different between the three groups. The mean AP equivalent dose was generally high in all groups, but the combination one showed the highest dose. There were more patients with psychotic disorders in the combination group (Table 2).

The total mean values of the various metabolic measures in our sample (Table 4) are all close to the normal upper range except for the BMI, which is higher than the standard value. All the metabolic measures in the FGA group were within normal and lower than the other two groups (Table 4). The available measures showed that patients maintained on SGA had higher mean weight and BMI compared to the FGA one and even those in the combination group. Both the SGA and combination groups showed higher systolic BP and TG compared to those in the FGA group. The fasting blood glucose was in the normal upper range, and there were no differences between the three groups. There were no enough measures on the glycosylated hemoglobin to make proper comparisons. The mean measures of the HDL and LDL levels in the three groups were within normal, and there were no significant differences between them (Table 4).

Elevated TG and obesity have been demonstrated in other retrospective studies of patients on AP when compared to the general population (Wysokinski et al. 2012). Other studies have shown that SGA had more effects on weight and BMI than FGA (De Hert et al. 2008). A retrospective study from Malaysia also showed similar mean metabolic measures in patients with ten years history of schizophrenia (Lee et al. 2018). The MetS is highly prevalent in the general populations of the Middle East, and the measures of the various risk factors of MetS vary considerably between countries and even in various studies from the same country (Ansarimoghaddam et al. 2018). A recent cross-sectional comparative study on the prevalence of MetS in patients maintained on AP in Qatar showed a higher level of BMI but similar measures of BP, fasting glucose, TG, and HDL (Hammoudeh et al. 2018) when compared to our retrospective study. A Saudi study conducted on psychiatric patients also reported higher BMI (28.6 ± 7.7 kg/m^2^), and fasting blood glucose (6.16 ± 2.48 mmol/L) when compared to ours (Alosaimi et al. 2017). There are no other studies from the countries of the Arabic Peninsula (usually have similar ethnicity distribution) that compared the effects of various AP on the metabolic measures. Most of the studies on MetS from this region and other Arab countries focused on patients with schizophrenia or other mental disorders rather than on the effects of AP per se. Such studies from UAE (Shahda et al. 2010), Kuwait (Akanji et al. 2007; Roshdy 2011), and Qatar (Bener et al. 2014) showed very similar patterns of increased BMI, lipid profile, and BP.

The proportion of subjects with abnormal metabolic measures in the whole sample is high ranging from about 21.5% for the TG and 52.5% for BP. Further, there were significantly more people with abnormal BP and BMI in the SGA and combination groups compared to the FGA one (Table 5). A Meta-analysis of the metabolic abnormalities in schizophrenia and other psychotic disorders reported similar meta-analytic rates for the abnormal glucose and HDL levels. However, our rate of abnormal TG was lower, and that of high BP was higher compared to this review (Mitchell et al. 2013). On the other hand, a cross-sectional study from Denmark on outpatients maintained on AP showed a lower proportion of low HDL, a similar rate for BP, and higher ones for TG and glucose (Krane-Gartiser et al. 2011) compared to our values. A comparative study from Venezuela showed that the type of AP or mental illness did not affect much the rate of the various metabolic abnormalities except for obesity that was higher in patients receiving olanzapine or clozapine (Baptista et al. 2011). Other retrospective population studies have demonstrated similar prevalence rates on obesity and MetS in patients maintained on AP and those from the general population where they also reported no differences in the prevalence of MetS between FGA and SGA (Santini et al. 2016). This study from Italy had a similar design as our study, also showed that the combination group showed more metabolic abnormalities than each class of AP alone. Furthermore, studies have also demonstrated a lack of effect of psychiatric illness or AP on the prevalence of DM type II when compared to that in the general population (Coulson et al. 2012). Another retrospective record review of the patients taking AP from Poland showed a similar prevalence of abnormal metabolic measures as in our study (Wysokinski et al. 2012). Again, there are very few studies from the Arabic countries that looked at the prevalence of the various metabolic measures and their association with the various AP. A study on patients with various psychiatric disorders from Saudi Arabia showed that the prevalence of the metabolic measures was not different when compared to those not taking AP. However, the intake of SGA was mainly associated with elevated BP (Alosaimi et al. 2017).

This variability in the changes of metabolic risk factors among the various studies could be attributed to several factors: genetics, ethnicity, age, gender, type of mental illness, type and dose of AP, duration of treatment, concomitant medications, family history of metabolic disorder, smoking, lifestyle, eating habits, etc. It is challenging to control for all these confounders, but some studies attempted to look at the various demographic and clinical variables that might be associated with metabolic abnormalities. Such studies have shown that women tend to show more weight gain on AP (Bener et al. 2014; Gates et al. 2016; Santini et al. 2016) than men but other studies showed mixed results in regard to gender (Hammoudeh et al. 2018; Lin et al. 2015; Susce et al. 2005). Concerning ethnicity, a previous study from Qatar showed that non-Arabs were more prone to develop metabolic changes in the population of Qatar (Hammoudeh et al. 2018), but other community studies worldwide showed no differences across race or ethnicity (Gates et al. 2016; Phillips et al. 2015). Several studies have also demonstrated that older age, long duration of treatment, and smoking are associated with more metabolic changes in patients with mental illness and receiving AP (Hammoudeh et al. 2018; Santini et al. 2016; Sweileh et al. 2012).

Monotherapy remains the recommended modality of treatment among schizophrenia patients (Sagud et al. 2013). In this regard, few studies assessed the relationship between polypharmacy and the severity of psychopathology. The valid measures in our sample showed that the frequency of delusions and hallucinations was the highest, especially in the combination group. A study from UAE also reported a high frequency of delusions (75.2%) and hallucinations (61.5%) in a similar population (Salem et al. 2008). Similar to our results, polypharmacy continues to be very common worldwide in patients with chronic mental disorders, especially those resistant to monotherapy or having multiple psychiatric disorders (John and Dragovic 2015; Yang et al. 2018). However, studies have shown that polypharmacy is associated with more side effects and MetS in psychotic patients (Jeon and Kim 2017). Also, academic interventions are effective in reducing unnecessary polypharmacy in patients with psychotic disorders (Mace and Taylor 2015). Auditing and standardizing computerized orders on patients maintained on AP have been shown to improve the recordings of the health measures but not the follow-ups or referral processes for those with abnormal measures (Barnes et al. 2015; Ross et al. 2018). Concerning the monitoring of patients on AP, low levels of metabolic screening have also been shown in other populations, e.g., a recent random retrospective review of 100 charts from Australia showed that none of the patients on AP were monitored every 3 months as recommended, and such monitoring decreased over one year (Ward et al. 2018). On the other hand, studies have shown that regular monitoring of patients maintained on SGA can significantly reduce the prevalence of MetS and the use of AP in general (Phillips et al. 2015; Reeves et al. 2017). Thus, closer clinical monitoring and administrative auditing are needed to improve the health outcomes of patients on AP in Qatar.

### Limitations

This study is the first to review the psychiatric records of the patients maintained on AP in the Arabic Gulf. Our findings might guide the foundation for future extensive or prospective studies and policy changes on monitoring patients maintained on AP. The sample size was adequate and covered patients with various psychiatric diagnoses and cultural backgrounds as per the unique structure of the population in Qatar. However, some disadvantages are worth discussing: First, being a retrospective study affects the inferential associations significantly and also increases the risk of having missing data. Second, there is no proper control group to compare the different measures obtained directly. Thus, more prospective studies with a control group are needed to assess the effects of AP on metabolic risk factors. Third, the heterogeneity of the sample regarding ethnicity, diagnosis, medication type, duration of illness, and treatment might hinder the generalizability of the results.

## Conclusions

Our results clearly showed that the SGA had more negative effects on BMI, TG, and BP in patients maintained on AP, all of which are known to increase the risk for cardiovascular diseases. These patterns are very similar to other international studies except for minor ethnic variations. We also demonstrated that polypharmacy is frequent in this population, where there is a lack of proper monitoring and adherence to the guidelines proposed. There is a clear need for more controlled prospective studies to guide the proper interventions. However, as the patterns of risk factors and polypharmacy are very similar to other worldwide studies, the international guidelines are the proper start to reinforce intensive monitoring of patients maintained on AP in Qatar.
